# Michael Ryan

**Published:** 1823-09

**Authors:** 


### OBITUARY.

DIED, a few months since, in Kilkenny, MICHAEL RYAN, M.D. for many years a most respectable practitioner in that city. He graduated at Edinburgh in 1784; since which period he published occasional essays on Influenza, Intermittent Fever, and on other subjects, in the early Numbers of the London Medical and Physical Journal. He also published a work on Asthma. There was no man for whose professional opinion the public entertained a greater regard. He was most assiduously industrious in pursuit of science, and an attentive observer of the various improvements in medicine, having perused every new literary medical publication, notwithstanding his extensive practice; and hence there were few men who formed a more correct diagnosis of disease.

There is a gentleman of the same name in that city at present, from whom an Essay appears in the present Number of this Journal.

